# Inferring Body Measurements from 2D Images: A Comprehensive Review

**DOI:** 10.3390/jimaging11060205

**Published:** 2025-06-19

**Authors:** Hezha Mohammedkhan, Hein Fleuren, Çíçek Güven, Eric Postma

**Affiliations:** 1 Department of Cognitive Science and Artificial Intelligence, School of Humanities and Digital Sciences, Tilburg University, 5037 AB Tilburg, The Netherlands; c.guven@tilburguniversity.edu (Ç.G.); e.o.postma@tilburguniversity.edu (E.P.); 2 Zero Hungerlab, Department of Econometrics and Operations Research, School of Economics and Management, Tilburg University, 5037 AB Tilburg, The Netherlands; fleuren@tilburguniversity.edu

**Keywords:** deep learning, convolutional neural network, automated anthropometry, artificial intelligence for nutrition

## Abstract

The prediction of anthropometric measurements from 2D body images, particularly for children, remains an under-explored area despite its potential applications in healthcare, fashion, and fitness. While pose estimation and body shape classification have garnered extensive attention, estimating body measurements and body mass index (BMI) from images presents unique challenges and opportunities. This paper provides a comprehensive review of the current methodologies, focusing on deep-learning approaches, both standalone and in combination with traditional machine-learning techniques, for inferring body measurements from facial and full-body images. We discuss the strengths and limitations of commonly used datasets, proposing the need for more inclusive and diverse collections to improve model performance. Our findings indicate that deep-learning models, especially when combined with traditional machine-learning techniques, offer the most accurate predictions. We further highlight the promise of vision transformers in advancing the field while stressing the importance of addressing model explainability. Finally, we evaluate the current state of the field, comparing recent results and focusing on the deviations from ground truth, ultimately providing recommendations for future research directions.

## 1. Introduction

**Figure 1:**
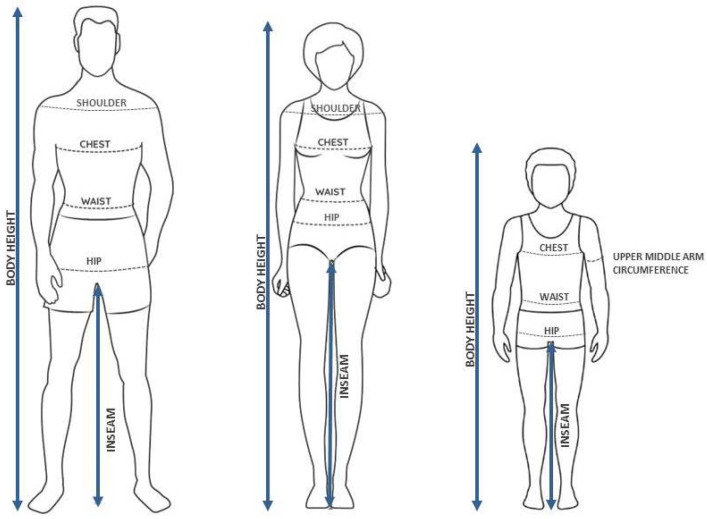
Illustration of commonly used body measurements of adults and children.

Anthropometric measurements are the systematic collection of data related to the physical dimensions and composition of the human body. These measurements are used to assess size, shape, and proportion, including attributes such as height, weight, body mass index (BMI), skinfold thickness, and circumferences of various body parts (e.g., waist, hip, chest, arms, etc.) [1]. To obtain anthropometric measurements, trained medical staff typically use non-stretchable tape measures, knee calipers, stadiometers, calibration weights, and weight scales [2]. However, while these traditional methods are considered the gold standard, especially for children (https://www.who.int/tools/child-growth-standards, accessed on 1 January 2025) they have disadvantages; to mention a few, manual anthropometric measurements (a) are heavily influenced by the measurement process and the condition of the human subject, (b) they require skilled personnel and calibrated equipment, and (c) they are time consuming, all of which affect the reproducibility of the results [3,4]. Figure 1 shows examples of standard body measurements collected from adult men, adult women and children.

In the last two decades, there has been a growing interest in advanced anthropometric research that uses 3D scanning technology to capture measurements from images of the subject [5]. 3D body scanning offers precise, reproducible, and fast anthropometric measurements, minimizing human error and inter-observer variability. However, 3D scanners are expensive, require specialized equipment and calibration, and can be affected by clothing artifacts and post-processing complexity [6]. Moreover, when used on children, only one out of three measurements is completed successfully; this is due to body movements or the inability of the software to extract shape outputs [7].

Height estimation from 2D images is a significant challenge in body measurement prediction and is one of the most prominent tasks in body measurement estimation. The primary difficulty arises from the ambiguity between an individual’s actual height and their distance from the camera, where variations in camera angle, focal length, and perspective distortion further complicate accurate estimation [8,9]. Without additional cues such as known reference objects or camera calibration, predicting height from 2D images becomes increasingly difficult, as the image alone does not provide sufficient depth information. Despite these challenges, height estimation has been a focal point due to its widespread applications.

The feasibility of estimating body measurements automatically from a 2D image has risen due to its various practical applications in healthcare, fashion, and fitness [10,11,12,13,14]. Combining traditional 2D image processing with modern deep-learning techniques enables scalable and automated large-scale applications, such as population surveys, that address the growing demand for AI-driven solutions in anthropometry. In particular, body measurements play a crucial role in detecting malnutrition and monitoring growth trends in young children, which motivates this review paper.

**Figure 2:**
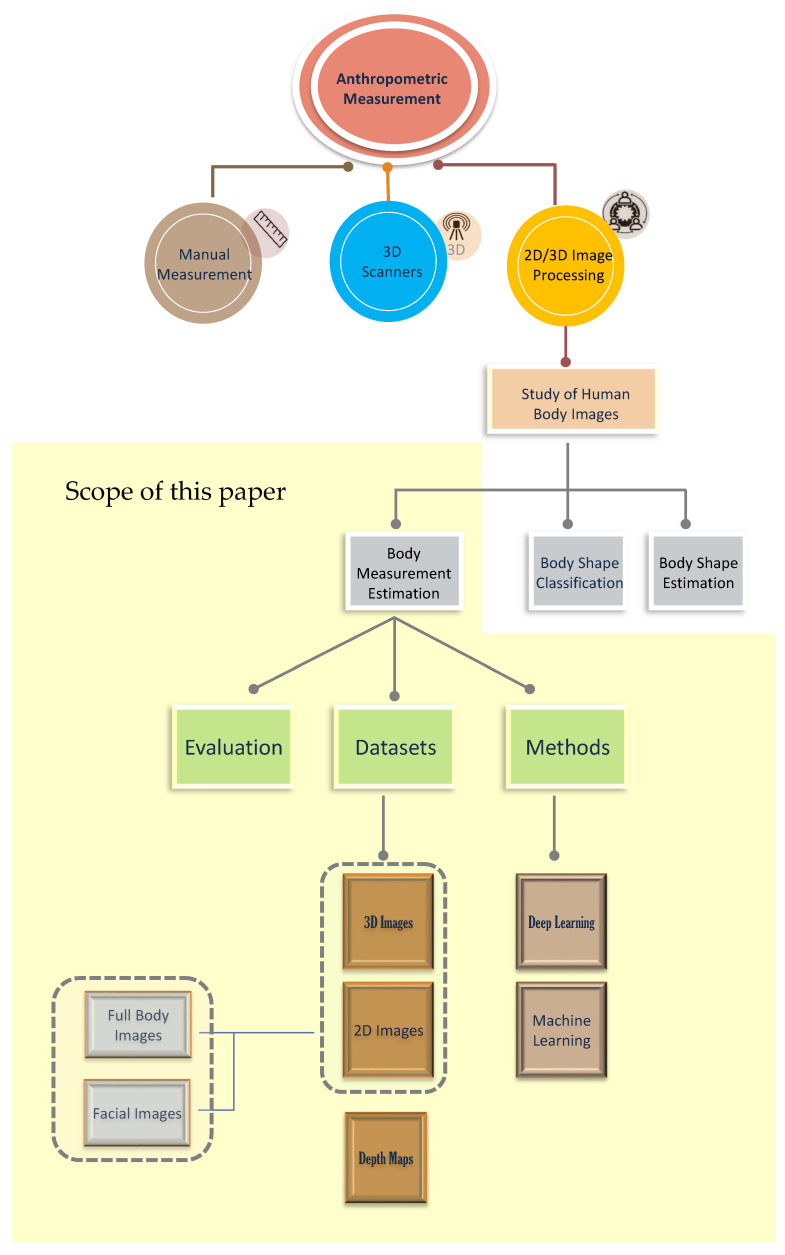
High-level overview of the literature review taxonomy.

Figure 2 illustrates the key topics identified in this review of the literature and demonstrates the process of reducing the literature into specific categories and focus areas. This taxonomy highlights the main themes and research directions explored in this review. Anthropometric measurement, the top of the taxonomy tree, is split into three types of measurement: manual, 3D scans, and 2D images. The review focuses on anthropometric measurement through 2D images, specifically on predicting BMI and body measurements.

## 2. Research Method

In this state-of-the-art review, the aim is to discuss the latest advancements, methods, and challenges in estimating body measurements from 2D images with machine-learning and deep-learning models, highlighting current trends and identifying key areas for future research. The methodology applied in performing this state-of-the-art review was adapted from Renner et al. [15] and previously published review papers on similar topics [16].

### 2.1. Data Sources and Search Strings

The primary studies were gathered from ACM Digital Library, Google Scholar, IEEE Explore, Springer Link, and Wiley InterScience. Different search queries were evaluated to retrieve the largest number of relevant papers; based on this criterion, the final search queries used for this research across all data sources were “Estimating body measurements from 2D images”, “Image-based body height estimations”, “BMI estimation from 2D images”, and “Dataset of human body measurements with images”. Among the various anthropometric attributes, height was selected as focal point because it is the most extensively studied body measure in computer vision and AI-based estimation tasks.

### 2.2. Inclusion Criteria

We define the inclusion criteria for papers and datasets included in our review. The three inclusion criteria used to select the reviewed articles are as follows.

i. Studies published in a peer-reviewed journal or conference proceeding.

ii. Studies written in English and published from 2017–2025.

iii. Experimental studies using computer vision techniques to estimate body measurements from 2D images.

The three inclusion criteria used to select the datasets are as follows.

i. Datasets must include at least 500 subjects.

ii. Datasets that include body measurements of its subjects, or can be preprocessed for measurements to be derived out of them.

iii. Datasets must be suitable for predicting body measurements from 2D images using ML and DL models. This can be done either directly using the dataset or with preprocessing, such as converting 3D bodies to 2D images.

### 2.3. Exclusion Criteria

The following criteria were used to exclude papers: (i) Duplicate papers found in data sources were removed. (ii) Exclude methodologically weak studies, such as those that lack solid validation methods. (iii) Studies that are based on anecdotal evidence and do not include performance metrics.

Although human shape and pose estimation, 3D body shape reconstruction, and body parameterization are relevant to body measurement estimation, they fall outside the core scope of this review, as illustrated in Figure 2. These fields often focus on broader applications, rely heavily on 3D data, and involve specialized methodologies distinct from our primary focus. Although we acknowledge their role in the creation and preprocessing of datasets—such as using parameterized models such as SMPL [17] for shape and pose estimation—our review does not go into these areas, other than explaining their relevance to body measurement estimation.

### 2.4. Selected Papers and Datasets

Once the inclusion and exclusion criteria were applied to the papers retrieved from the data sources, 47 papers met the inclusion criteria for this review, excluding papers used in the discussion. Table 1 provides a list of these studies and their publication year, these are the papers reviewed for methods and evaluation criteria.

## 3. Three Types of Body-Shape Estimation Tasks

The study of human body images spans numerous research fields with even broader potential applications. We identify three computational tasks related to making inferences about body shape: body measurement estimation [28,34,59], body shape and pose estimation [60,61,62], and body shape classification [63].

For this review, we focus primarily on the first task, body shape estimation, as it is the core concept under examination. The last two tasks provide foundational insights relevant to body measurement estimation. Therefore, we start by discussing these two tasks before turning to the core task of this review.

**Human shape and pose estimation** (HPE) is the task of estimating the 3D positions and orientations of body joints and bones from 2D images and videos [56] or 2D joint locations [57,64]. Notable HPE methods, such as OpenPose [57] and Humanoid [65], detect key points on a 2D plane for computational simplicity, although they lack depth information. Both OpenPose and Humanoid can integrate multiple camera views or depth sensors to estimate a z-coordinate for each joint [29]. Although both methods provide estimates of relevant body measurements, they do not include measurements of musculature, such as head circumference and waist circumference. In contrast, the Skinned Multi-Person Linear (SMPL) model [17] does include such measurements. SMPL is a foundational in model-based HPE, representing the body as a parameterized mesh. In SMPL, shape and pose are represented by separate groups of parameters. The SMPL model has been extended to represent more details of the body. For instance, SMPL-X captures anatomical details, clothing, and joint silhouettes for added realism [48]. The relevance of HPE methods for this review is that models such as SMPL provide a suitable foundation for inferring body measurements. For example, waist circumference is encoded within the shape component of the parameterized SMPL model.

**Figure 3:**
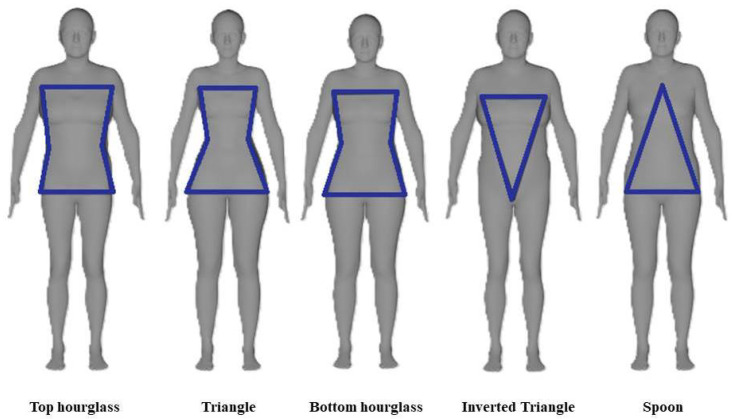
Examples of common body shapes used in apparel fitting.

**Body shape classification** is the task of assigning human bodies to different proportion categories. Figure 3 illustrates the most common classes of body shapes: top hourglass, triangle, bottom hourglass, inverted triangle, and spoon. These classifications are widely used in apparel fitting [46], and automating this process using images can improve size recommendations in online shopping [21]. The most effective approaches take advantage of traditional machine learning or deep learning, typically using body measurements, rather than raw images, as input [41,66]. Key measurements such as height [30], shoulder width, and waist circumference [66] are commonly used for classification.

While body shape classification and direct body measurement estimation serve different purposes, they share key computational principles, including keypoint extraction, pixel-to-centimeter conversion, and proportionality analysis, making it highly relevant to this review paper. These shared foundations suggest that techniques developed for body shape classification can be adapted for precise body measurement estimation. For example, using height to estimate waist circumference, determining a person’s height by referencing objects in the background, or by applying pixel-to-centimeter conversions [30].

**Body measurement estimation** is the core task of this review and involves the direct prediction of numerical measurements of different body characteristics, such as height, waist circumference, and head circumference. These measurements can be used to infer weight, and the combination of height with weight can be used to calculate the body mass index (BMI) [67] by means of Equation (1).

(1) $$\mathrm{BMI}=\frac{\mathrm{weight}}{{\mathrm{height}}^{2}},$$

with weight expressed in kg and height expressed in meters. Body measurement estimation from 2D images greatly benefits many domains, such as health, fashion, and fitness. Body measurements are often used to determine the nutritional status of both adults and children, as BMI is the main health indicator for adults. For children, the combination of height and weight as a function of age is the standard indicator of growth in children under the age of 5 [68,69].

Although there have been extensive reviews on human shape and pose estimation [31,32,47,70,71] and body shape classification [30,72], there is a notable gap in reviews of papers on body measurement estimation from 2D images, as it remains a relatively underexplored area [36]. A recent paper presented a comprehensive overview of digital anthropometry for medical applications [6], but did not cover image-based anthropometry estimation. In the remainder of this paper, we aim to fill in this gap in the literature and provide a comprehensive overview of this domain. Our interest lies in the ability to extract body measurements of children from images, and to use them as a support tool for the monitoring of child growth, as proposed in [10,42].

## 4. Body Measurement Estimation Methods

Estimating body measurements purely from 2D images is still a very challenging task despite major developments in computer vision. The reason for this is that a 2D image does not provide sufficient information to infer the 3D shape of the body. In other words, inferring the object shape from a single 2D image is underconstrained [73]. Depth images [42,74] or 3D scans could make inferring object’s shape easier, but require special equipment and may be computationally demanding [20].

Recent advances in computer vision, mainly through deep-learning techniques such as convolutional neural networks (CNNs) have enabled accurate predictions of body measurements from images [11,37,49,50]. Vision transformers have been widely used for human pose estimation in recent years [75], but their application in human body measurement estimation seems to be underexplored. CNNs are deep-learning models that process spatially (or temporally) structured data, such as images. CNNs for visual tasks use convolutional layers to automatically learn hierarchies of visual features, making them particularly effective for image classification, object detection, and feature extraction. CNNs have demonstrated exceptional performance in computer vision, both for visual regression and classification tasks. In the context of this review, the regression task for estimating body measurements is of primary interest.

### 4.1. Traditional Machine-Learning Methods

We refer to machine-learning (ML) algorithms that do not rely on deep-learning algorithms as traditional machine learning. Focusing on regression, rather than classification, examples of (traditional) ML algorithms are: linear regression, logistic regression, support vector regression, k-nearest neighbor regression (k-NN), and elastic net regression [76]. Although this review focuses primarily on image-based methods for body measurement estimation, we also include selected statistical baselines where relevant, particularly when they directly compete with deep-learning approaches. For example, Bartol et al. [36] proposed a linear regression baseline to predict 15 body measurements using only height and weight as input. Trained on synthetic SMPL mesh data (BODY-fit), the model achieved surprisingly competitive results. They were benchmarked against several deep-learning-based methods also provided in Section 6, demonstrating that traditional regression models can perform comparably to, or better than, deep models. A major limitation of this approach is that height and weight have to be provided, which limits the applications of this method.

In general, ML-based body measurement estimation from images relies on features extracted from the image because ML algorithms can only deal with low-dimensional inputs. ML models, such as logistic regression or support vector regression [77] are trained on features that summarize image properties or represent task-relevant cues. Three examples of such cues are (i) the distance between the person and the camera, (ii) an object in the background with known dimensions [52,55], or (iii) the height of a region of interest (bounding box) generated by a human-body detection algorithm [22].

Given that many anthropometric measurements are not statistically independent, they can also be used as cues. As a case in point, features such as arm length or waist circumference are effective cues to infer other body dimensions such as stature and weight, or vice versa [3,23,36,53].

Features can also be obtained using statistical methods. Principal component analysis (PCA) [78] can be applied to images or 3D scans to create low-dimensional features (i.e., principal components) for body measurement estimation. For 3D meshes of body shapes, typically the first principal component represents stature, the second waist circumference, and so forth [10].

#### Combining Traditional Machine-Learning and Deep-Learning Methods

Some researchers integrate traditional ML techniques with deep learning in hybrid models that take advantage of both structured and learned features [49]. The hybrid models typically rely on a late-fusion approach where feature integration occurs after separate representation learning. Jin et al. [37] proposed a dual-branch regression framework in which one branch uses pose-estimation techniques to extract anthropometric features such as joint distances, while the other applies deep learning to extract body contour features from full-body images. These independently processed features are combined using a Gaussian process regression model, i.e., late fusion. A similar late-fusion approach was applied in a recent infant body measurement study [19], which estimated recumbent length using smartphone photographs and a reference card of known scale. Anthropometric landmarks were detected and combined with image-based measurements to produce length estimates. Although limited methodological details are provided, this study demonstrates the adaptation of hybrid techniques to pediatric applications. Another relevant late-fusion example is the SHAPY framework [38], which combines RGB images, semantic shape attributes, and metric measurements through separate encoder branches. These features are fused in a regression module to predict SMPL body shape parameters. While SHAPY is designed for 3D shape reconstruction rather than direct anthropometric measurement, it reflects a similar ate fusion architecture using multi-modal inputs. These studies rely on predefined anatomical features without exploring automated techniques like RFE, SHAP, or LASSO. This highlights a gap in the literature regarding the optimization of feature combinations in hybrid models.

### 4.2. Deep-Learning Methods

**Figure 4:**
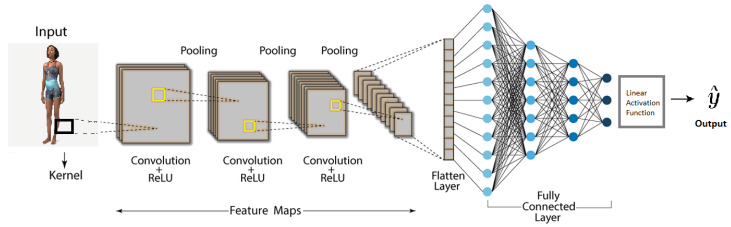
Illustration of a basic CNN for a classification task.

End-to-end Learning is a widely used approach, where a single model is responsible for both feature extraction and final prediction. Figure 4 is an illustration of an end-to-end deep-learning architecture. The input is an image that is processed by several convolution and pooling layers. After successful training, the convolution layers incorporate feature detectors that support the regression task at hand. The pooling layers perform a local averaging operation to ensure that the dimensionality is gradually lowered with each subsequent layer.

In one of the few papers that includes child data [42] applied an end-to-end approach was applied using depth images obtained from video-to-depth image conversion to predict height, primarily to assess if the height is too low for the age (i.e., stunting). Although this study achieved commendable results within an acceptable range, the limited availability of data for research and replication curtails its potential impact.

Similarly, Velesaca et al. [28] presented a methodology for human height estimation using a stereo vision system. Their approach combined the YOLO v7 and MediaPipe models and demonstrated efficient real-time human detection with better performance and lower hardware requirements, and an MAE below 1.0 cm in the height estimation. Despite the promise shown in controlled environments, the reliance on substantial data and computing power to train deep CNN models raises challenges, as the model must learn features directly from complex input data, potentially constrained by data quality and diversity.

Transfer learning is a common strategy in the domain of visual tasks. CNNs that are pre-trained on large image datasets in order to solve task X are subsequently trained on a related but different task Y for which only a small dataset is available. Transfer learning works because all visual tasks are based on common “front-end” visual features. As a consequence, the lower levels of pre-trained CNNs incorporate features that are common to all visual tasks. In contrast, the higher levels of these CNNs tend to be more task-specific.

**Figure 5:**
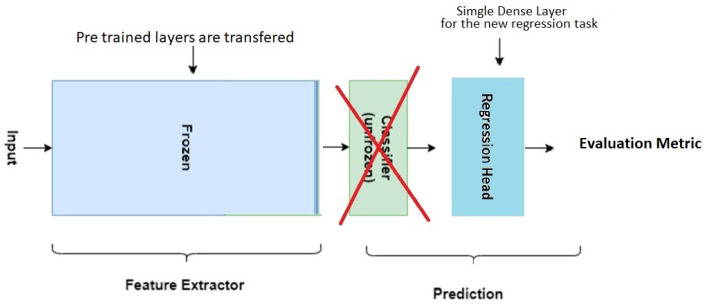
Illustration of transfer learning where the convolution and pooling layers of pre-trained CNN are frozen, i.e., the adaptive parameters are fixed, forming a feature extractor (left rectangle). The extracted features are then passed to a newly initialized fully connected layer or multiple layers (right rectangle), which is trained on a task-specific dataset to adapt the model to a new problem domain. This approach allows the model to leverage prior knowledge while reducing the need for large amounts of task-specific labeled data.

**Figure 6:**
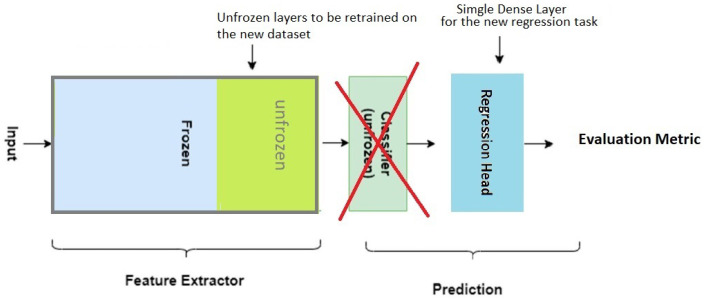
Illustration of a variant of transfer learning in which only the bottom part of the feature extractor is frozen.

Figure 5 and Figure 6 illustrate two common transfer-learning approaches. In Figure 5, the left rectangle represents the convolution and pooling layers of a CNN, pre-trained on an image-classification task. These layers act as a generic feature extractor for any visual task. The center rectangle (with a cross) represents the original dense layers that map these features on the proper classes and, therefore, called a classification head. In transfer learning, the classification head is replaced by randomly initialized dense layers and trained on the new task. In our context, it becomes a regression head.

There are many variants of transfer learning, one of which is illustrated in Figure 6. Instead of freezing the entire feature extractor, only its bottom part is frozen. This gives the CNN more flexibility in tuning the top (more task-specific) layers of the feature extractor. Other variants of transfer learning replace freezing by a gradient of learning rates in which the bottom layers of the pre-trained feature extractor have very small learning rates, and subsequent layers have increasing learning rates.

Transfer learning has been widely used in visual tasks, including image-based body measurement tasks such as image-based BMI prediction [51,54]. Ichikawa et al. [43] estimated body weight using a CNN to predict body weight from chest and abdominal CT scans using transfer learning. Mohammedkhan et al. [10] adopted another transfer-learning approach to estimate the characteristics of body shapes from images of virtual human body shapes. They used CNNs pretrained on another visual task to estimate waist circumference and body height.

### 4.3. Limitations of CNN and the Rise of Alternative Architectures

Although convolutional neural networks (CNNs) remain central to body measurement estimation tasks, they exhibit several limitations. CNNs require large annotated datasets, which makes them less effective in low-resource or domain-specific contexts. They also have high computational demands and limited interpretability, important considerations for applications in healthcare, mobile environments, or real-time settings. Furthermore, decisions made by CNNs are hard to explain and understand; they require extensive research to understand their decisions. Recent advances in vision architectures may offer promising alternatives. Vision transformers (ViTs) provide global attention mechanisms that can model long-range spatial relationships in body structure, potentially improving robustness to pose variation and occlusion [25]. ViTs were recently explored and compared in terms of body measurement estimation performance with powerful CNN models [18]. For this particular case, CNNs outperformed ViTs. Still, given their potential, ViTs should be further explored in the context of body measurement estimation. Diffusion models, known for their strong performance in generative vision tasks [33], have been applied to 3D shape reconstruction and localized editing [26], as well as human pose and shape refinement in images [27]. However, their potential for body measurements estimation remains largely unexplored.

## 5. Body Measurement Estimation Datasets

In the field of predicting body measurements from 2D images, datasets play a crucial role in training and evaluating machine-learning and deep-learning models. A well-curated dataset not only provides the necessary inputs (images) and outputs (body measurements) for model training, but also ensures that the models generalize well to unseen data. The quality and richness of the dataset, including accurate labeling, diverse samples, and high resolution, directly affect the performance and robustness of predictive models [44,58]. In this section, we provide an overview of the commonly used datasets for predicting anthropometric measurements from images. Table 2 provides a high-level comparison of widely used publicly available datasets, focusing on size, number of subjects, application for which the dataset was originally curated, whether it provides body measurements, inclusion of children, and format of the dataset. While this review aims to identify 2D image datasets, we have also included 3D image datasets, as they can be converted to 2D by means of projection.

Our selection focuses on datasets that feature full-body or facial images labeled with more than 500 subjects and several thousand labeled images, which are crucial for advancing machine-learning and deep-learning tasks. Datasets not originally designed for body measurement estimation were selected based on their pose in the image; a standard A pose makes it easier to perform statistical shape analysis and extra body measurement values in arbitrary units [10], while those with different poses are more difficult to process. Several smaller datasets, such as Human Body in the Wild [38], provide valuable information for training deep neural networks; they fall outside the scope of this review because they contain less than 500 unique subjects or fall short according to our inclusion criteria outlined in Section 2.2.

As shown in Table 2, the IMDb-23K dataset [50] remains a significant resource, offering publicly available celebrity images with height annotations derived from the original IMDb database. However, its reliance on in-the-wild images introduces variability in lighting, pose, and background, which can impact data quality and model performance.

The MORPH-II dataset [85], a longitudinal face image dataset, is constrained by limited ethnic diversity, predominantly representing individuals of African and European descent, with minimal inclusion of other ethnic groups. The Visual-body-to-BMI dataset [49] offers a range of body types but lacks representation of infants, children under 5 years old, and adolescents under 16 years, limiting its suitability for pediatric malnutrition and growth studies.

The CAESAR dataset, which contains 3D human body shapes, is restricted by commercial licensing and excludes individuals under 18 years old. Moreover, it primarily comprises subjects from North America and Europe, limiting its applicability to broader, more diverse populations. These demographic and age biases across datasets underscore the challenges of model generalization and fairness in anthropometric estimation tasks.

To address some of these gaps, the recently released ARAN dataset [18] provides a GDPR-compliant collection of multi-view full-body images and detailed anthropometric measurements from 512 children aged 16 to 98 months. They did include important procedural adaptations during data collection: children who could not stand unassisted or were in critical condition were excluded to prevent harm and reduce the burden to healthcare staff. Separately, there is related work such as SMPLify-KiDS [86], which introduces methodological adaptations for markerless 3D motion tracking tailored to children’s unique body shapes and movements. Although this line of research focuses on motion tracking and full-body shape reconstruction rather than direct body measurement estimation from images, their approach could be adapted to the curation of larger children datasets to include infants and account for their variability in motion.

### Preprocessing and Data Augmentation

Datasets used for body measurement estimation often require significant preprocessing to ensure consistency and improve model performance. For datasets originally containing 3D scans (e.g., CAESAR, BODY-fit), standard practice involves projecting 3D meshes into 2D images from multiple standardized viewpoints, such as front, side, and back. These projections use fixed camera parameters to simulate controlled imaging environments [11].

Further image preprocessing typically includes resizing images to a uniform resolution compatible with CNN architectures (commonly 224 × 224 pixels), normalization of pixel intensities by subtracting the dataset mean and scaling to a standard range, and cropping or alignment to focus on the human body region, minimizing background interference [18,50]. Such standardization reduces variability and facilitates more stable training.

Data augmentation techniques are widely adopted to enhance model robustness against environmental variations. Common augmentations include random rotations, horizontal flips, color jittering, scaling, and noise addition [23]. Augmentation is particularly important for in-the-wild datasets with uncontrolled lighting, poses, and backgrounds, as it helps mitigate overfitting and improves generalization. Synthetic data generation techniques, particularly those based on generative adversarial networks (GANs), have proven effective in increasing dataset size and diversity for 3D human body reconstruction [87]. However, current GAN-based methods do not produce precise body measurements, limiting their direct application in domains requiring accurate anthropometric data [88]. Future advances in synthesis precision could enhance their suitability for body measurement estimation tasks.

## 6. Evaluation

A standard approach for evaluating model performance in predicting body measurements primarily relies on regression metrics, with mean absolute error (MAE) being the most commonly used. Validation typically follows one of two protocols: (a) K-fold cross-validation on the training set—commonly 5-fold or 10-fold—after an initial 80/20% train-test split [19,35,43], or (b) external dataset testing, where the trained model is assessed on a separate dataset to evaluate generalization [36]. However, external dataset evaluation remains underutilized in this domain and should be more widely adopted. Another notable limitation across the literature is the general absence of formal statistical significance testing when comparing model performances. For instance, the ARAN study [18] examines correlations and uses a linear baseline to assess the relationship between measurements and image features, but it does not perform statistical hypothesis tests on the model results.

Most studies rely on within-dataset testing and report results using different datasets, making fair comparison between models difficult. Further, details such as the number of training subjects, input modality (e.g., 2D RGB, depth, silhouette), and model type are often reported inconsistently or omitted entirely.

To address these issues and provide a more structured overview, Table 3 summarizes a representative selection of studies on body measurement estimation. It reports MAEs for height (H), weight (W), waist circumference (WC), and head circumference (HC) where available. In addition to the performance metrics, we include a qualitative estimate of computational difficulty (Comp.), based on model architecture, dataset structure, and input format.

**Figure 7:**
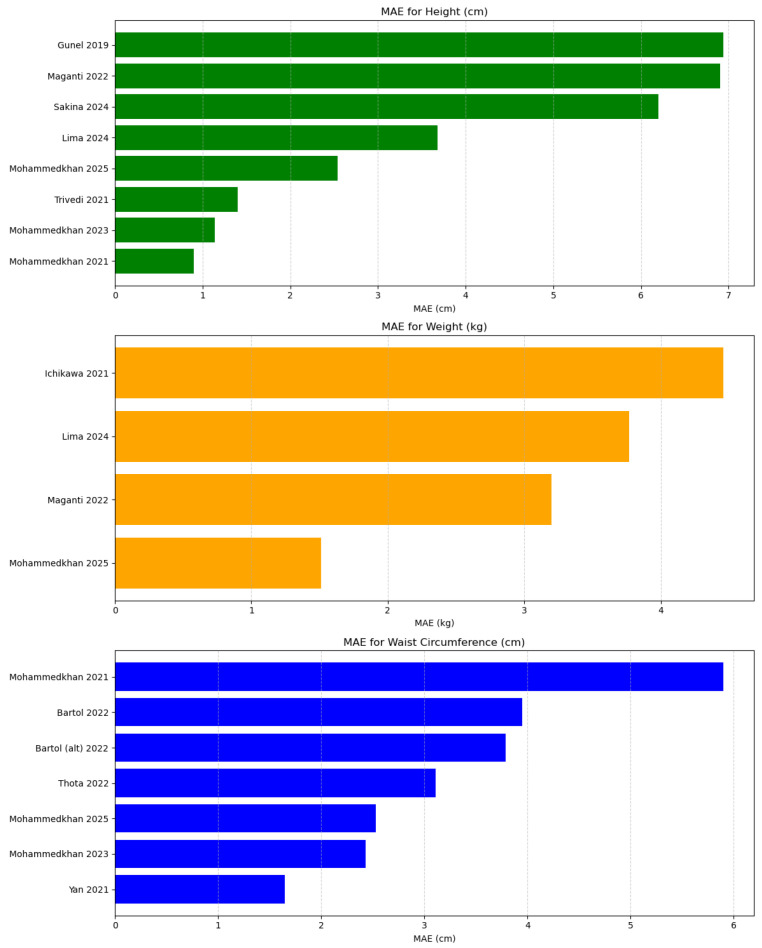
Comparison of mean absolute error (MAE) across studies for height, weight, and waist circumference prediction [10,11,18,23,24,36,39,40,42,43,45,50].

The table and Figure 7 shows that structured datasets—such as synthetic 3D body renders or depth images collected under controlled clinical conditions—tend to yield substantially lower MAEs in height estimation tasks. For instance, Mohammedkhan et al. [10] report an MAE of 0.90 cm using 3D synthetic meshes. This performance can be attributed to the standardized camera angles, lack of occlusion, and the precise alignment of virtual body models, which eliminate noise from background clutter, clothing, or pose variability. Similarly, Trivedi et al. [42], working with depth images of children captured in a clinical setting, achieve an MAE of 1.40 cm. Depth images offer an advantage by directly encoding body geometry and minimizing the influence of surface texture, lighting, or clothing.

In contrast, models trained on unconstrained, in-the-wild RGB images—such as those used by Gunel et al. [50] and Sakina et al. [23]—report higher MAEs in the range of 6–7 cm. These higher errors are likely due to the increased variability in pose, background, lighting, and clothing, as well as the absence of camera calibration. In these settings, the model must learn to generalize across diverse image conditions, making precise body measurement estimation significantly more challenging.

Weight estimation follows a similar trend: Maganti et al. [39], using silhouette images, achieve a MAE of 3.2 kg, while Yilmaz and Achanta [59] report 9.8 kg using unconstrained RGB images. In the domain of circumference estimation, Yan et al. [45] achieve the lowest errors (e.g., 16.5 mm for waist), using clean silhouette data and a CNN-based architecture.

A recent addition is the ARAN dataset [18], which provides labeled full-body images of children in clinical settings. Using DenseNet121, the authors report MAEs of 2.54 cm for height, 1.51 kg for weight, 25.3 mm for waist, and 15.2 mm for head circumference, demonstrating feasibility in child-specific applications. This is the first paper to perform these experiments on children bodies, and while their results are promising, if we compare these to results achieved by [10,42], we can assume that the performance is affected by lightning, camera calibration, and clothes in the ARAN dataset.

External dataset testing, where a model trained on one dataset is evaluated on an entirely separate dataset, is a more robust approach to assessing generalization. For example, Bartol et al. [36] trained a simple linear regression model on synthetic SMPL meshes (BODY-fit) and tested it on the ANSUR dataset containing manually collected real-world measurements. To ensure comparability, they applied mesh scaling to align the predicted body height and ground truth, enabling meaningful evaluation across datasets with different sources and units. Although this method does not use any images or deep-learning techniques, their inclusion in this review is crucial as it provides a strong non-image baseline to contextualize the value (and cost) of using visual models. A similar protocol is provided by Mohammedkhan et al. [18], where a linear baseline based on the correlation between the body measurements provides a baseline for the performance of the deep-learning models.

The computational difficulty of body measurement estimation methods depends primarily on model architecture, input data modality, and dataset size and complexity. Simple linear regression models using tabular features like height and weight impose minimal computational requirements and are classified as low difficulty. Large convolutional neural networks trained on extensive, unconstrained in-the-wild RGB image datasets require significant computational resources, resulting in high difficulty. Medium difficulty corresponds to CNNs operating on noisy or less-structured RGB images with moderate model complexity. Methods leveraging small CNNs trained on clean synthetic 3D renders demand low computational effort. Deep CNN architectures such as DenseNet121 applied to multi-view clinical pediatric datasets require moderate computational resources, placing them in the medium difficulty category. The higher the computational difficulty, the more expensive it is in terms of training cost, as larger computers would require and use of electricity.

## 7. Discussion

In this section, we discuss the three crucial components of body measurement estimation: Methods, Datasets, and Evaluation.

### 7.1. Methods

Among the various approaches for predicting body measurements from images, convolutional neural networks (CNNs)—whether custom-built, pre-trained, or integrated into hybrid pipelines—have demonstrated strong predictive performance. However, their robustness remains a critical challenge. Many models fail to generalize well beyond their training conditions, with performance deteriorating under variations in camera calibration, image resolution, and environmental factors. Studies comparing predictions with and without access to standard camera parameters have highlighted these limitations [10,11]. Furthermore, the prevalent use of limited or homogeneous datasets restricts model generalizability. As a result, models that perform well on curated benchmarks may struggle with diverse poses, body types, or real-world imagery [24].

To date, CNNs remain the dominant architecture in body measurement estimation. However, recent work comparing CNNs with vision transformers (ViTs) suggests that this may not remain the case. While CNNs currently outperform ViTs in this domain [18], trends in other fields show increasing parity—or even superiority—of ViTs in various vision tasks [90]. Advances in ViT design, such as optimized patch size, structured tokenization, or improved positional encoding, may shift the balance. Notably, hybrid CNN-ViT models have shown promising results in domains like medical imaging [91], suggesting a similar potential for anthropometric estimation. Finally, diffusion models, while generative in nature, can potentially be adapted for regression-based body measurement estimation by conditioning on specific metrics and introducing loss functions such as mean absolute error (MAE). However, precise real-world measurement prediction requires pixel-to-metric calibration, which is a challenge across many models, not just diffusion-based ones. Notably, Liu and Wang (2025) proposed an uncertainty-guided diffusion model for 3D human pose estimation that improved accuracy in difficult regions, an approach that could inspire similar adaptations for anthropometric use [92].

**Proposed solution:** To improve robustness and generalizability, future models should be trained on more diverse, in-the-wild datasets that reflect a wide range of camera setups, body poses, and demographic attributes, including racial and ethnic diversity. Such dataset heterogeneity is crucial to mitigating bias and ensuring consistent performance across global populations. Additionally, exploring alternative or hybrid architectures, such as diffusion models, ViTs, and CNN-ViT combinations [93], may better capture complex visual features and lead to more adaptable and accurate anthropometric prediction models.

### 7.2. Datasets

Despite significant advancements in data collection for human body measurement estimation, existing datasets still exhibit notable limitations that are connected to those of the methods described above. Three primary concerns are the lack of ethnic diversity, the under-representation of children, particularly those under the age of 16, dataset validity, and dataset quality. In what follows, we discuss each of these concerns.

Lack of ethnic diversity: Many widely used datasets, such as the MORPH-II dataset [85], predominantly consist of individuals of African and European descent, with limited representation from other ethnic groups. This lack of diversity can introduce biases in model predictions, reducing generalizability when applied to populations not adequately represented in the training data. Similarly, the CAESAR dataset, which contains 3D human body scans, is primarily composed of individuals from North America and Europe, limiting its applicability to global populations. Ensuring a wider demographic representation is critical for the equitable deployment of body measurement technologies.

Under-representation of children: The absence in datasets of representative samples of children, particularly infants and individuals under 16 years of age, is a concern. Most datasets, including the Visual-body-to-BMI dataset [49] and CAESAR, do not include young individuals, restricting the ability to study growth patterns and malnutrition in early childhood. Even the extensive IMDbv23K dataset [50] is composed solely of celebrity images, which does not accurately reflect demographics. In addition, the dataset lacks crucial metadata on children’s body measurements. This omission is of particular concern for healthcare and nutritional studies, where early-life developmental measurements are essential for deciding on effective intervention strategies.

Dataset validity: Many of the reviewed datasets were originally designed for purposes other than body measurement prediction, such as pose estimation, facial analysis, or ergonomic assessment. As a result, they may lack the specific labeling accuracy or pose diversity required for precise body measurement estimation. In some cases, key attributes, such as camera calibration data or ground-truth height and weight labels, are missing, limiting their utility for certain predictive tasks. Additionally, several high-quality datasets, such as CAESAR, are commercially restricted, posing barriers to reproducibility and large-scale model development. The lack of standardized evaluation protocols and cross-dataset benchmarks further complicates the comparison of model performance and generalizability.

Dataset quality: Finally, to ensure high-quality and reliable predictions, datasets must consist of high-resolution 2D images or 3D body scans that comprehensively capture individuals of all ages and ethnic backgrounds. These datasets should also provide full-body coverage from multiple viewpoints to account for variations in body posture, perspective distortions, and measurement accuracy.

**Proposed solution:** While a single, universally comprehensive dataset may be unrealistic, curated datasets that reflect the diversity and variability in specific target populations are essential. In practice, an effective alternative is to develop datasets and methods tailored to particular population segments, such as children, specific ethnic groups, or individuals in clinical settings. While these models may not generalize beyond their intended demographic, they can achieve high accuracy within it. Enriching the global dataset landscape should be a collective effort among researchers, with complementary datasets that, together, ensure fair and robust representation across a wide range of human populations and use cases.

### 7.3. Evaluation

Comparing the performances of different models for body measurement estimation presents several challenges due to variations in datasets, applications, and intended use cases. Each dataset may differ in terms of sample diversity, imaging conditions, and ground-truth annotations, making direct performance comparisons difficult. Furthermore, models designed for distinct applications, such as healthcare, fashion, and fitness, have different priorities regarding accuracy, computational efficiency, and ethical considerations. To establish a fair and consistent evaluation framework, standardized metrics and benchmarking protocols should be developed. These should facilitate model comparisons within the same application domain and across datasets with similar characteristics. Without such standards, reported model performances may lack reproducibility and real-world applicability. An example of a good standard is the SMART methodology [94]. This methodology is adopted for malnutrition detection and defines an explicit criterion for height prediction, i.e., estimates should have an MAE of no more than 1.2–1.4 cm [42].

The variability in images further compounds the complexity of evaluating body measurement estimation models. A typical image used for body measurement estimation may depict individuals in diverse poses, wearing different clothing, and being captured under various environmental conditions. Factors such as camera distance, angle, viewpoint, and lighting introduce inherent ambiguities that affect model performance. These external factors can lead to inconsistencies in the accuracy of the prediction and create additional challenges in defining a universal evaluation criterion. However, these characteristics are highly dependent on data collection procedures and ethical constraints around that procedure.

**Proposed solution:** Despite the challenges mentioned earlier, some recent works offer promising examples. Mohammedkhan et al. [18] include a linear baseline using correlations between measurements to contextualize deep model performance. These efforts suggest that future benchmarking protocols should incorporate both non-image baselines and correlation-aware evaluations that account for age, sex, and size stratification. We recommend that such protocols define a common set of parameters, including: image resolution (e.g., 256 × 256 or 512 × 512), pose specifications (e.g., frontal vs. side view, recumbent for infants), reference object or scale calibration (e.g., inclusion of a known-length marker), lighting and background conditions, demographic variables (e.g., age groups, stature percentiles) and dataset size. These elements would allow researchers to evaluate performance more consistently across datasets and models, and enable them to perform more meaningful comparisons between deep-learning approaches and simpler statistical baselines.

### 7.4. Explainable AI

Explainability is crucial in many domains, particularly in medical and healthcare applications [95], where understanding how a model makes decisions is essential for trust and regulatory approval. In contrast, industries such as fashion and gaming may not require the same level of transparency, allowing for more flexibility in model selection and deployment. This disparity highlights the trade-off between model interpretability and performance across use cases. Convolutional neural networks (CNNs), which dominate current approaches to body measurement estimation, are often criticized for their lack of interpretability. They function as “black boxes”, making it difficult to trace how specific inputs influence specific outputs. Techniques such as saliency maps, Grad-CAM, and SHAP have been proposed to improve transparency in deep models [96,97].

**Proposed solution:** The most promising approaches to explainability in body measurement estimation are those that create heat maps or similar visualizations to allow practitioners to verify the processed body locations that give rise to the estimation. A recent example is the effective combination of Grad-CAM and LRP, which achieves enhanced visual explainability [98]. No studies have applied these or similar explainability tools to body measurement estimation from 2D images, making this a clear and actionable research gap. However, recent works on explainability in human-body-related tasks, such as pose estimation using Group Shapley Value [99] and activity recognition using SHAP and Grad-CAM [100] demonstrate the feasibility of applying these techniques to structured prediction problems involving human representations. These models could be adapted to enhance transparency in anthropometric prediction tasks, particularly in assessing health and nutritional status, where interpretability is essential. Further, as AI systems for body estimation move toward real-world deployment, particularly in health contexts, standardized explainability metrics analogous to MAE or RMSE should be considered for regulatory and ethical evaluations. This topic warrants further interdisciplinary investigation.

### 7.5. Readiness for Real-World Deployment

Despite impressive results in controlled lab settings, many body measurement models encounter significant challenges when applied to real-world environments. Applications such as virtual try-ons, fitness apps, and tell medicine platforms must contend with variations in pose, clothing, lighting, and background, which often cause model performance to degrade, as discussed in Section 6.

One of the potential future applications of body measurement estimation models is that they can be used in a dynamical setting to check and correct estimation errors. For instance, we envisage a future digital twin system that maps the inferred body measurements on a 3D model of the human (child) under consideration that is adapted in response to the estimates. In turn, the 3D model provides an effective prior for subsequent estimates when, for instance, changes in viewpoint or pose affect the estimates. Such a digital twin approach would improve the quality of body measurement estimations in health and virtual garment applications.

The main challenges to achieve such a digital-twin approach are latency, accuracy, and privacy. Current software and hardware are too slow to achieve the seamless dynamical interaction required for a digital twin system. Given the rapid development of deep-learning software and hardware, it is to be expected that the latency problem will be solved within a few years. Although the current accuracy achieved by body measurement estimation may fall short for one-shot estimation, the incorporation in a dynamical feedback system may elevate it to an acceptable level.

Furthermore, consumer-grade devices typically lack built-in camera calibration, further exacerbating issues related to geometric consistency. Addressing these challenges requires the development of domain adaptation techniques, extensive robustness testing, and models capable of functioning reliably under diverse and uncontrolled imaging conditions.

Nevertheless, image-based body measurement is already being deployed in selected commercial sectors. In the fashion industry, services like MTailor [101] have demonstrated real world viability by using a single smartphone camera image to measure body dimensions for custom clothing. MTailor claims that its measurements are 20% more accurate than those taken by professional tailors, though these figures are proprietary and not independently validated. Similarly, virtual try-on systems in e-commerce and gaming platforms increasingly use parametric models (e.g., SMPL-based avatars) to personalize clothing fit or character design. These systems often prioritize visual plausibility over numerical precision and typically operate within pre-calibrated or synthetic environments [102].

In contrast, applications in healthcare and clinical nutrition impose higher standards of accuracy, robustness, and explainability. For example, the SMART guidelines for malnutrition detection specify that height estimation tools must have an MAE below 1.2–1.4 cm to be considered viable for field deployment [94]. Our review shows that only a few methods meet this threshold, and even then, only under constrained conditions (e.g., synthetic or clinical datasets), this threshold would vary in adults and would be measured by the BMI ratio. Furthermore, medical applications require interpretable and auditable models, which deep-learning tools often fail to provide due to their black-box nature. These limitations make current systems insufficient for clinical deployment without further development in explainability, calibration, and cross population generalization.

### 7.6. Ethics, Bias, and Privacy

Body measurement estimation from images inherently involves sensitive biometric data. Surprisingly, few papers explicitly address the ethical implications of collecting, processing, or deploying these models. Researchers must ensure that datasets are collected with informed consent, especially when children or vulnerable populations are involved.

An exception is the ARAN dataset [18], which provides a clear example of ethically grounded data collection for children. The data were collected with parental or guardian consent, approved by an institutional review board, and subjected to anonymization procedures to protect participant privacy. This serves as a strong precedent for ethical practices in pediatric applications. More of these data anonymization techniques and procedures should be considered in future pipelines. The widespread use of public datasets also calls for clarity on licensing, as many are shared under CC-BY or similar licenses, but that does not prevent misuse unless contextual restrictions are honored. Ethical development also requires a discussion of potential misuse, such as surveillance, discrimination, or body shaming.

Achieving demographic fairness is another persistent challenge. The scarcity of globally diverse, high quality datasets limits the ability to assess and mitigate algorithmic bias. This highlights a growing tension between the need for representative data and the constraints imposed by privacy, legal, and logistical concerns, which requires further interdisciplinary investigation. There are system-level tools and automated frameworks that exist to monitor and flag biases in real-time model predictions in health tracking wearables, such as IBM’s AIF360 [103], but their development and integration specifically for body measurement estimation remain nascent.

## 8. Limitations and Conclusions

This review provided a comprehensive synthesis of methods and datasets for inferring body measurements from 2D images, covering classical machine learning, CNN-based approaches, and recent developments such as vision transformers (ViTs). While this offers a solid foundation for understanding current trends and technical progress, several limitations and future directions should be highlighted.

- Scope of applications: The review did not cover real-time or closed-loop systems such as digital twins, biomechanical or thermal simulations, or textile interaction modeling. These applications require edge-compatible architectures, low-latency inference, and integrated feedback mechanisms. Their implementation lies beyond the scope of current image-based body measurement models and remains a fertile ground for future research.
- Demographic fairness and policy: Achieving fairness across gender, ethnicity, and body types remains a critical challenge. The lack of globally representative datasets significantly constrains algorithmic generalization. Although this review addressed fairness conceptually, it did not cover tools for real-time bias monitoring or policy-enforced auditing. Emerging work on sample reweighting and fairness toolkits like AIF360 shows promise but has yet to be adapted for regression-based anthropometric prediction. The development of domain-specific fairness metrics and bias detection tools is urgently needed.
- Limitations related to cross-sector technologies and integration with Industry 5.0: Although our review is centered on AI-based methods for inferring body measurements from 2D images, primarily for child growth monitoring and nutritional assessment, it is important to acknowledge the growing relevance of body measurement estimation in Industry 5.0 initiatives, particularly in the garment sector. Recent research has shown how technologies such as digital human modeling (DHM), digital twins, and E-Libraries are being integrated to enable virtual sizing, customer-specific fit prediction, and sustainable production pipelines [104,105,106]. For example, e-body libraries can store user-specific measurements and 3D avatars, which are matched with garments using AI to reduce returns and enhance personalization. These systems often rely on body measurement data inserted by the user, but can also be inferred from images or scans, sometimes incorporating AI-based methods like the ones reviewed here. However, our paper does not address the engineering or implementation of such closed-loop systems. Instead, we focus on the methodological underpinnings of AI models for body measurement prediction. We see these industrial applications as complementary but distinct, and suggest that future interdisciplinary work could explore how AI models developed for healthcare may also serve as foundational components in garment technology platforms or digital twin systems.

Despite these limitations, three key opportunities emerge for advancing the field. First, future work should systematically evaluate the suitability of emerging architectures, such as ViTs, diffusion models, and hybrid CNN-ViT networks, for anthropometric regression tasks. Second, the creation of ethically collected, demographically diverse datasets remains essential for enhancing model robustness and fairness. Third, consistent evaluation protocols must be established to enable valid cross-study comparisons and reproducibility.

Given the privacy-sensitive nature of anthropometric data, especially in pediatric or healthcare contexts, research should also explore privacy-preserving learning strategies. One promising avenue is federated learning [107], which enables collaborative model training without sharing raw data. Although not yet applied in the reviewed studies, such methods could help address data access constraints while preserving participant anonymity. Finally, long-term integration of body measurement estimation into Industry 5.0 systems, including smart manufacturing, digital wardrobes, and human–AI co-design platforms, will require cross-disciplinary collaboration and policy engagement. While these directions remain outside the scope of this review, they represent a critical next phase in transforming technical progress into practical, equitable, and human centered solutions.

## Figures and Tables

**Table 1:** Selected studies based on search strings and inclusion criteria.

Author(s)	Title	Year
Mohammed Khan et al. [18]	ARAN: Age-restricted Anonymized Dataset of Children Images and Body Measurements	2025
Chua et al. [19]	Exploring the Use of a Length AI Algorithm to Estimate Children’s Length from Smartphone Images in a Real-World Setting	2024
Wang et al. [20]	From prediction to measurement, an efficient method for digital human model obtainment.	2024
Park et al. [4]	Efficient Model-Based Anthropometry under Clothing Using Low-Cost Depth Sensors.	2024
Pang et al. [21]	Learning Visual Body-shape-Aware Embeddings for Fashion Compatibility.	2024
Fadllullah et al. [22]	Automatic human height measurement system based on camera sensor with deep-learning and linear regression analysis.	2024
Sakina et al. [23]	A multi-factor approach for height estimation of an individual using 2D image.	2024
Lima et al. [24]	A scale-equivariant CNN-based method for estimating human weight and height from multi-view clinic silhouette images.	2024
Takeda, Toshiaki et al. [8]	Calibration-Free Height Estimation for Person	2024
Pereira and Hussain [25]	A review of transformer-based models for computer vision tasks: Capturing global context and spatial relationships	2024
Potamias et al. [26]	Shapefusion: A 3D diffusion model for localized shape editing	2024
Okuyama et al. [27]	DiffBody: Diffusion-Based Pose and Shape Editing of Human Images	2024
Velesaca et al. [28]	Deep Learning-based Human Height Estimation from a Stereo Vision System.	2023
Kim et al. [29]	Human pose estimation using mediapipe pose and optimization method based on a humanoid model.	2023
Trotter et al. [30]	Human body shape classification based on a single image.	2023
Chen et al. [31]	2D Human Pose Estimation: A Survey	2023
Kulkarni et al. [32]	PoseAnalyser: A Survey on Human Pose Estimation	2023
MohammedKhan et al. [11]	Image-Based Body Shape Estimation to Detect Malnutrition	2023
Ji et al. [33]	DDP: Diffusion Model for Dense Visual Prediction	2023
Abadi et al. [34]	Digital Image Processing for Height Measurement Application Based on Python OpenCV and Regression Analysis	2022
Jin et al. [35]	Attention Guided Deep Features for Accurate Body Mass Index Estimation	2022
Bartol et al. [36]	Linear Regression vs. Deep Learning: A Simple Yet Effective Baseline for Human Body Measurement	2022
Jin et al. [37]	Estimating Human Weight from A Single Image	2022
Choutas et al. [38]	Accurate 3D Body Shape Regression using Metric and Semantic Attributes	2022
Maganti et al. [39]	Height and Weight Estimation of an Individual from Virtual Visuals	2022
Thota et al. [40]	Estimation of 3D Body Shape and Clothing Measurements from Frontal- and Side-View Images	2022
Mohammed Khan et al. [10]	Predicting Human Body Dimensions from Single Images: A First Step in Automatic Malnutrition Detection	2021
Foysal et al. [41]	SmartFit: Smartphone Application for Garment Fit Detection	2021
Trivedi et al. [42]	Height Estimation of Children Under Five Years Using Depth Images	2021
Ichikawa et al. [43]	A Deep-Learning Method Using Computed Tomography Scout Images for Estimating Patient Body Weight	2021
Varga [44]	No-Reference Image Quality Assessment with Convolutional Neural Networks and Decision Fusion	2021
Yan et al. [45]	Silhouette Body Measurement Benchmarks	2021
Tinsley et al. [5]	Digital Anthropometry via Three-Dimensional Optical Scanning: Evaluation of Four Commercially Available Systems	2020
Yu et al. [46]	Body Shape Classification of Korean Middle-Aged Women Using 3D Anthropometry	2020
Zheng et al. [47]	Deep Learning-Based Human Pose Estimation: A Survey	2020
Pavlakos et al. [48]	Expressive Body Capture: 3D Hands, Face, and Body from a Single Image	2019
Jiang [49]	Body Weight Analysis from Human Body Images	2019
Gunel et al. [50]	What Face and Body Shapes Can Tell Us About Height	2019
Haritosh et al. [51]	A Novel Method to Estimate Height, Weight and Body Mass Index from Face Images	2019
Dhikhi et al. [52]	Measuring Size of an Object Using Computer Vision	2019
Ashmawi et al. [53]	FitMe: Body Measurement Estimations Using Machine Learning Method	2019
Liu et al. [3]	Single Camera Multi-View Anthropometric Measurement of Human Height and Mid-Upper Arm Circumference Using Linear Regression	2018
Dantcheva et al. [54]	Show Me Your Face and I Will Tell You Your Height, Weight and Body Mass Index	2018
Kriz et al. [55]	Determination of a Person’s Height from Images Using a Known Object Size	2018
Martínez et al. [56]	Pose Estimation and Tracking of Non-Cooperative Rocket Bodies Using Time-of-Flight Cameras	2017
Cao et al. [57]	Realtime Multi-Person 2D Pose Estimation Using Part Affinity Fields	2017
Yim et al. [58]	Enhancing the Performance of Convolutional Neural Networks on Quality Degraded Datasets	2017

**Table 2:** Comparison of datasets for body measurement prediction: summary of dataset content, characteristics, and ethnic composition.

Dataset	# Images	# Subjects	Application	Labels	Children	Face/Body/Scan	Demographic Coverage
**CAESAR [79]**	N/A	2400	Ergonomics, apparel, healthcare	Yes	No	3D Scans	North American and European
**ANSUR [80]**	N/A	13,000	Military, ergonomics, apparel	Yes	No	3D Scans	US Military, mostly white
**Human3.6M [71]**	3.6 m	11	Pose estimation, 3D modeling	No	No	Body pose	Limited
**TC^2^ [81]**	N/A	Variable	Apparel, ergonomics	Yes	Unknown	3D Scans	Not specified
**SCANative [82]**	N/A	Unknown	Indigenous population anthropometry	Yes	Unknown	No	Indigenous populations (Canada)
**CANDAT [83]**	N/A	Unknown	Healthcare, nutrition, growth studies	Yes	Yes	No	Not specified
**NHANES [84]**	N/A	Unknown	Public health, nutrition, growth studies	Yes	Yes	No	Diverse U.S. population
**IMDB-23K [50]**	23,000	12,104	Age, gender prediction, height analysis	Yes (Height)	No	Face/Full body	Diverse western celebrities
**MORPH-II [85]**	55k academic– 202k commercial	55,000	Age estimation, demographic studies	Yes (Age, Gender)	No	Facial Images	77% black, 19% white, rest other
**Body-Fit [45]**	NA	4149	Body measurement estimation	Yes (16 body measurements)	No	Silhouette images	Not specified
**Image-to-BMI [37]**	4189	3000	BMI, gender, age, height, and weight estimation	Yes	No	Full body images	Diverse (unknown distribution)
**ARAN** [18]	2048	512	Pediatric growth and malnutrition monitoring	Yes (Height, weight, waist, head circ.)	Yes	Body (multi-view)	Kurdish

**Table 3:** Unified benchmark of body measurement estimation using CNNs and regression methods. Tasks: H = Height, W = Weight, WC = Waist Circ., HC = Hip Circ., TC = Thigh Circ., CC = Chest Circ., WR = Wrist Circ. MAE = Mean Absolute Error. Comp. Diff. = Computational Difficulty (L = Low, M = Medium, H = High).

Publication	Dataset	H	W	WC	HC	TC	CC	WR	Comp. Diff.
		(cm)	(kg)	(mm)	(mm)	(mm)	(mm)	(mm)	
Mohammedkhan et al. [10]	CAESAR renders	0.90	–	59	–	–	–	–	M
Trivedi et al. [42]	CGM custom data	1.40	–	–	–	–	–	–	M
Lima et al. [24]	Botanic solution [89]	3.68	3.77	–	–	–	–	–	M
Sakina et al. [23]	Image-to-BMI	6.20	–	–	–	–	–	–	M
Maganti et al. [39]	Custom celebrity RGB	6.90	3.20	–	–	–	–	–	M
Gunel et al. [50]	IMDB-23K	6.94	–	–	–	–	–	–	H
Ichikawa et al. [43]	CT scans	–	4.46	–	–	–	–	–	H
Yilmaz & Achanta [59]	IMDB-23K	–	9.80	–	–	–	–	–	H
Thota et al. [40]	Synthetic 3D renders	–	–	31.1	24.4	–	29.4	–	M
Bartol et al. [36]	BODY-fit	–	–	39.5	28.0	16.0	30.3	2.7	L
Bartol et al. (alt) [36]	ANSUR	–	–	37.9	21.6	17.0	29.1	4.4	L
Mohammedkhan et al. [11]	CAESAR + AGORA	1.14	–	24.3	10.3	–	–	–	M
Yan et al. [45]	Silhouette dataset	–	–	16.5	14.0	14.6	22.0	4.5	M
**Mohammedkhan et al. [18]**	ARAN	2.54	1.51	25.3	15.2	–	–	–	M

## Data Availability

Not applicable.
